# Supplementary material for: Pomegranate Woody Mycobiota Associated with Wood Decay
Source: J Fungi (Basel). 2025 Mar 26;11(4):254. doi: 10.3390/jof11040254 (PMC12028045; doi:10.3390/jof11040254)
Supplement: Supplementary file 1 [file jof-11-00254-s001.zip › jof-3504623-supplementary.pdf]

Supplemental Materials

Sample	Primer ID	Illumina overhang adapter sequences	*Index tag*	Original primer
1	1Forw	5′TCGTCGGCAGCGTCAGATGTGTATAAGAGACAG	ACACAC	GCATCGATGAAGAACGCAGC3′
	1Rev	5′GTCTCGTGGGCTCGGAGATGTGTATAAGAGACAG	ACACAC	TCCTCCGCTTATTGATATGC3′
2	2Forw	5′TCGTCGGCAGCGTCAGATGTGTATAAGAGACAG	ACAGCA	GCATCGATGAAGAACGCAGC3′
	2Rev	5′GTCTCGTGGGCTCGGAGATGTGTATAAGAGACAG	ACAGCA	TCCTCCGCTTATTGATATGC3′
3	3Forw	5′TCGTCGGCAGCGTCAGATGTGTATAAGAGACAG	GTCTCT	GCATCGATGAAGAACGCAGC3′
	3Rev	5′GTCTCGTGGGCTCGGAGATGTGTATAAGAGACAG	GTCTCT	TCCTCCGCTTATTGATATGC3′
4	4Forw	5′TCGTCGGCAGCGTCAGATGTGTATAAGAGACAG	GCGCTC	GCATCGATGAAGAACGCAGC3′
	4Rev	5′GTCTCGTGGGCTCGGAGATGTGTATAAGAGACAG	GCGCTC	TCCTCCGCTTATTGATATGC3′
5	5Forw	5′TCGTCGGCAGCGTCAGATGTGTATAAGAGACAG	ACATGT	GCATCGATGAAGAACGCAGC3′
	5Rev	5′GTCTCGTGGGCTCGGAGATGTGTATAAGAGACAG	ACATGT	TCCTCCGCTTATTGATATGC3′
6	6Forw	5′TCGTCGGCAGCGTCAGATGTGTATAAGAGACAG	ACGACG	GCATCGATGAAGAACGCAGC3′
	6Rev	5′GTCTCGTGGGCTCGGAGATGTGTATAAGAGACAG	ACGACG	TCCTCCGCTTATTGATATGC3′
7	7Forw	5′TCGTCGGCAGCGTCAGATGTGTATAAGAGACAG	ATATCG	GCATCGATGAAGAACGCAGC3′
	7Rev	5′GTCTCGTGGGCTCGGAGATGTGTATAAGAGACAG	ATATCG	TCCTCCGCTTATTGATATGC3′
8	8Forw	5′TCGTCGGCAGCGTCAGATGTGTATAAGAGACAG	CTCGCA	GCATCGATGAAGAACGCAGC3′
	8Rev	5′GTCTCGTGGGCTCGGAGATGTGTATAAGAGACAG	CTCGCA	TCCTCCGCTTATTGATATGC3′

Table S1: Sequences of primer pairs used for the 8 experimental units (sample) showing, separately, the adapter sequences for the Illumina system, the indexed tag and the sequences of the universal ITS3 and ITS4 (original primer) are shown separately.

**EFFECT**	**SS**	**DF**	**MS**	**F**	**ProbF**
Isolate	24.012	1	24.012	30.836	0.012 *
Error	2.336	3	0.779		
Total	26.349	7			

Table S2: ANOVA results conducted on length (cm) of browning caused by the positive control, *N. parvum* (ER 2123) inoculated on the pomegranate detached twigs in humid chamber in 2019.

**EFFECT**	**SS**	**DF**	**MS**	**F**	**ProbF**
Isolate	66.701	1	66.701	22.102	0.018 *
Error	9.054	3	3.018		
Total	75.755	7			

Table S3: ANOVA results conducted on length (cm) of browning caused by the positive control, *N. parvum* (ER 2123) inoculated on the pomegranate detached twigs in water in 2019.

**EFFECT**	**SS**	**DF**	**MS**	**F**	**ProbF**
Isolate	107.311	1	107.311	32.871	0.011 *
Error	9.794	3	3.265		
Total	117.105	7			

Table S4: ANOVA results conducted on length (cm) of browning caused by the positive control, *N. parvum* (ER 2123) inoculated on the pomegranate plants in 2020.

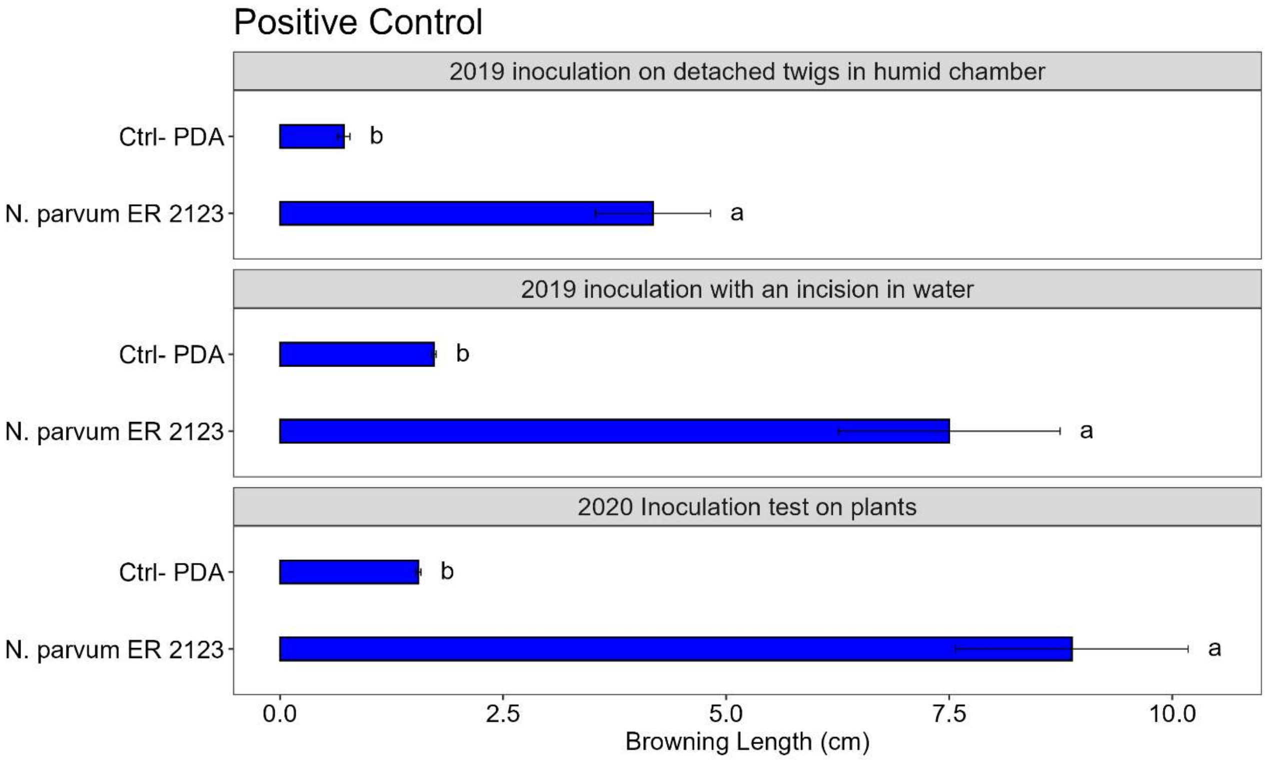

Figure S1: Bar plot showing the average browning lengths (cm) caused by inoculations with the positive control Neofusicoccum parvum (ER 2123) across three inoculation methods. The letters above the bars indicate significant statistical differences between treatments (Tukey's HSD, $p < 0.05$). Different letters indicate significant differences based on One-Way ANOVA results at $p < 0.05$. Error bars represent the standard error of the mean (4 replicates).
